# Circulating levels of carbamylated protein and neutrophil extracellular traps are associated with periodontitis severity in patients with rheumatoid arthritis: A pilot case-control study

**DOI:** 10.1371/journal.pone.0192365

**Published:** 2018-02-02

**Authors:** Chihiro Kaneko, Tetsuo Kobayashi, Satoshi Ito, Noriko Sugita, Akira Murasawa, Kiyoshi Nakazono, Hiromasa Yoshie

**Affiliations:** 1 Division of Periodontology, Department of Oral Biological Science, Niigata University Graduate School of Medical and Dental Sciences, Niigata, Japan; 2 General Dentistry and Clinical Education Unit, Niigata University Medical and Dental Hospital, Niigata, Japan; 3 Department of Rheumatology, Niigata Rheumatic Center, Shibata, Japan; Keio University, JAPAN

## Abstract

**Objectives:**

An interrelationship between rheumatoid arthritis (RA) and periodontitis has been suggested due to their common pathogenic mechanisms. Protein carbamylation and neutrophil extracellular traps (NETs) formation have been shown to be related to autoimmune conditions, including RA, but their association with periodontitis has not been elucidated. Therefore, we assessed whether or not circulating levels of carbamylated protein (CarP) and NETs are associated with periodontitis severity and influenced by periodontal treatment.

**Methods:**

We conducted a retrospective case-control study that included 40 patients with RA and periodontitis, 30 patients with periodontitis, and 43 systemically and periodontally healthy controls to assess the circulating levels of CarP and NETs and rheumatologic and periodontal conditions. The same assessments were also performed in 22 patients with RA and periodontitis after 2 months of periodontal treatment, including oral hygiene instruction and full-mouth supragingival scaling.

**Results:**

Patients with RA and periodontitis showed significantly higher serum levels of CarP and NETs than the control group (P = 0.04 and P < 0.001, respectively). The serum levels of CarP and NETs were significantly correlated positively with the mean values of probing depth (P = 0.01 and P = 0.007, respectively) and clinical attachment level (P = 0.007 and P = 0.001, respectively) in the 40 patients with RA and periodontitis. Multiple logistic regression analyses also revealed significantly positive associations between the serum levels of CarP and NETs and moderate to severe periodontitis (P = 0.03 and P = 0.001, respectively). Furthermore, periodontal treatment significantly decreased the serum levels of CarP and NETs in patients with RA and periodontitis (P = 0.03 and P = 0.02).

**Conclusion:**

The circulating levels of CarP and NETs are associated with periodontitis severity and influenced by periodontal treatment in patients with RA.

## Introduction

Rheumatoid arthritis (RA) is a chronic inflammatory joint disease that causes damage to the cartilage and bone as well as disability [1]. The prevalence of RA in the world's adult population is approximately 0.5% to 1.0% [2]. Periodontitis is also a chronic inflammatory disease that results from dysbiosis of oral microbiota, causing the loss of the connective tissue and alveolar bone support of the teeth [3]. The worldwide prevalence of severe periodontitis, as indicated by deep periodontal pockets of ≥ 6 mm, is 10% - 15% in adults [4]. A number of studies have suggested an epidemiological, serological, and clinical association between RA and periodontitis, which may be bi-directional in that RA affects the manifestation of periodontitis and, conversely, periodontitis is a risk factor for RA [5–7]. In addition, treatments for both diseases influence each other in that periodontal treatment improves the clinical and biochemical measures for RA [8], and targeted therapy for RA ameliorates the periodontal condition [9]. These observations imply that RA and periodontitis are interrelated through their common pathogenic mechanisms.

The majority of patients with RA (50% - 70%) are seropositive for autoantibodies against anti-cyclic citrullinated peptide (CCP) as well as for those against immunoglobulin G (IgG) known as rheumatoid factor (RF) [1]. Of these, anti-CCP autoantibodies play an important role in the pathophysiology of RA and thus constitute a specific biomarker that can be detected years before the onset of RA [1, 10]. In addition to citrullination, carbamylation, another post-translational modification of proteins, also produces autoantibodies that can trigger inflammatory conditions, including RA [11, 12]. Recent evidence has suggested that anti-carbamylated protein (CarP) antibodies may be useful as a diagnostic marker for RA but are independent of anti-CCP antibodies [13, 14]. Experimental arthritis studies have also shown that the appearance of anti-CarP antibodies in the sera precedes the onset of RA [15, 16]. Recently, the presence of CarP was also demonstrated in inflamed periodontal tissue from patients with mild to moderate periodontitis [17]. These findings led to the hypothesis that protein carbamylation in patients with RA may also be shared by periodontitis. However, no study has evaluated the relevance of circulating CarP levels to periodontitis or its severity.

Protein carbamylation is catalyzed by myeloperoxidase (MPO) released from neutrophils [11]. The critical role of neutrophils in innate immunity is well recognized by the phagocytosis of bacteria and formation of neutrophil extracellular traps (NETs). NETs are web-like structures that consist of chromatin backbones, histones, and antimicrobial proteins, including MPO [18]. Previous studies have indicated increased levels of NET generation and anti-CCP antibody production in the serum and synovial fluid from RA patients [19, 20]. These observations suggest that NETs may be a potential source of citrullinated proteins that are associated with autoimmune responses. However, other studies have also reported that MPO levels were elevated in the sera, salivary, and gingival crevicular fluid of patients with periodontitis compared with control individuals [21, 22]. NET formation is also detected in supragingival biofilms, gingival crevicular exudate, and inflamed periodontal tissue [23, 24]. These findings suggest that MPO-associated NETs may be a potential source of CarP in relation to periodontal inflammation, findings that conflict with those of another study [25]. It is therefore clinically important to determine whether or not circulating NETs levels are relevant to periodontitis and its severity.

The aim of the present study was to compare the circulating levels of CarP and NETs among patients with RA and periodontitis, patients with periodontitis only, and systemically and periodontally healthy controls. In addition, the relationship between the circulating levels of CarP and NETs and periodontitis severity was evaluated. Furthermore, the effect of periodontal treatment on the same biomarkers was also assessed in patients with RA and periodontitis.

## Materials and methods

### Ethics statement

The present study was approved by the Ethics Committee of Niigata University (Permit Number 2017–0114) and Niigata Rheumatic Center (Permit Number 2017–009), which is in accordance with the Declaration of Helsinki and conformed to the STROBE (strengthening the reporting of observational studies in epidemiology) guidelines [26]. All participants provided their written informed consent to participate in the present study.

### Study design and population

**Fig 1:**
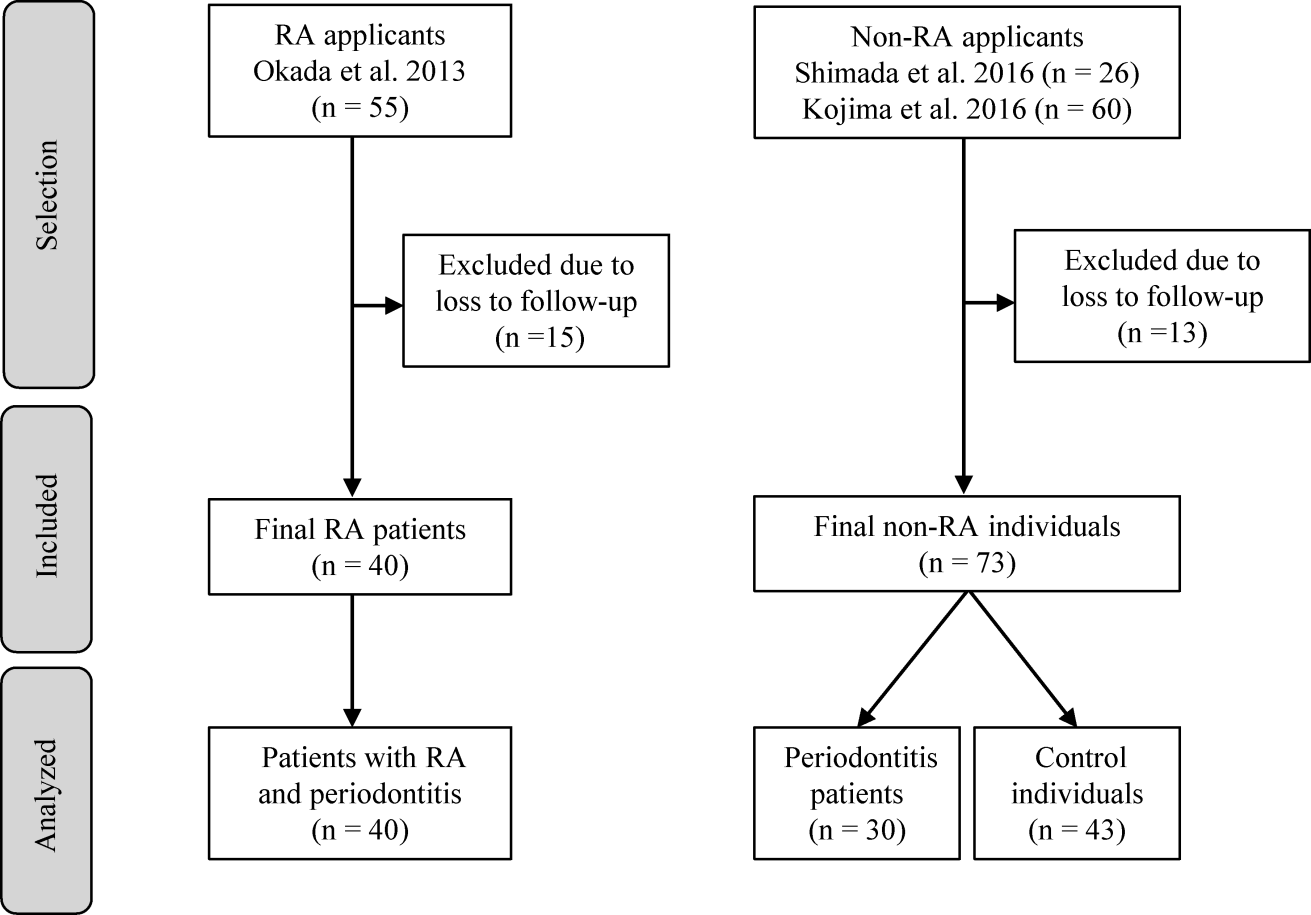
Flowchart of the study participant selection.

A retrospective case-control study was conducted at Niigata University Hospital and Niigata Rheumatic Center. The study applicants were 55 patients with RA and 86 with no signs of RA or other systemic diseases who were retrieved from 3 previous studies [27–29] (Fig 1). In brief, all applicants had undergone medical interviews that assessed their smoking status (never-smoker, former-smoker, or current-smoker), rheumatologic and periodontal examinations, and blood collections. Rheumatologists and periodontists evaluated each applicant independently and were blinded from each other regarding the rheumatologic and periodontal condition. All age, gender, smoking, rheumatologic, and periodontal data were extracted from the databases of the Niigata Rheumatic Center and Niigata University Hospital. RA was diagnosed by three licensed rheumatologists (SI, AM, and KN) according to the 1987 revised classification criteria of American Rheumatism Association [30] as well as the 2010 RA classification criteria of the American College of Rheumatology and European League Against Rheumatism [31]. Periodontitis was defined by one licensed periodontist (TK) based on the criteria of the Centers for Disease Control and Prevention (CDC)/American Academy of Periodontology (AAP) [32]. The eligibility criteria for patients with RA and those with periodontitis were having ≥ 15 teeth; the absence of diabetes mellitus, as assessed by HbA1c ≥ 6.5% and fasting plasma glucose ≥ 126 mg/dl [33]; the absence of pregnancy; and no history or the absence of any periodontal and antibiotic treatment within the previous 3 months. The eligibility criteria for the controls included systemically healthy individuals without periodontitis. Of these applicants, 15 for RA and 13 for non-RA who were lost to follow-up were excluded. Thus, the final analyzed study population included 40 patients with RA and periodontitis (RA+periodontitis group), 30 with periodontitis only (periodontitis group), and 43 control individuals (control group) (Fig 1).

Furthermore, of the 40 patients in the RA+periodontitis group, 22 had been randomly assigned to receive periodontal treatment including oral hygiene instruction and full-mouth supragingival scaling with ultrasonic instruments and were thus retrieved from a previous study [27]. All of the rheumatologic and periodontal data obtained before and after 2 months of periodontal treatment (baseline and reassessment) were extracted from the databases as mentioned above.

### Periodontal examination

Clinical periodontal data were collected by one calibrated and licensed periodontist (TK) as previously described [28], including the number of teeth present, O'Leary's plaque control record [34], bleeding on probing, probing depth, and clinical attachment level. The bleeding on probing, probing depth and clinical attachment level were measured at six sites around each tooth with a Williams probe (Hu-Friedy, Chicago, IL, USA). The reproducibility of the clinical measurements was calculated based on the intra-examiner intraclass correlation coefficient of 0.90. Periodontitis cases were defined as follows [32]: severe periodontitis, the presence of ≥ 2 proximal sites with a clinical attachment level of ≥ 6 mm (not on the same tooth) and at least 1 proximal site with a probing depth of ≥ 5 mm; moderate periodontitis, the presence of ≥ 2 proximal sites with a clinical attachment level of ≥ 4 mm, or ≥ 2 proximal sites with a probing depth of ≥ 5 mm (not on the same tooth); mild periodontitis, the presence of ≥ 2 proximal sites with a clinical attachment level of ≥ 3 mm, and ≥ 2 proximal sites with a probing depth of ≥4 mm (not on the same tooth) or 1 site with a probing depth of ≥ 5 mm. Participants who did not meet these criteria were considered not to have periodontitis.

### Rheumatologic examination

Clinical rheumatologic data were obtained by three licensed rheumatologists (SI, AM, KN) and stored in the Niigata Rheumatic Center database, which included the disease duration and activity of RA, tender and swollen joint counts, and the patient’s general assessment of his/her condition scored on a visual analog scale (VAS). The disease activity of RA was determined with the Disease Activity Score including 28 joints using C-reactive protein (DAS28-CRP), which was calculated based on 4 components: the tender joint count, swollen joint count, VAS, and CRP measurements [35]. The serological rheumatologic data included anti-CCP IgG titers that were determined by an enzyme-linked immunosorbent assay (ELISA) with a commercially available kit (Medical & Biological Laboratories, Co., Ltd. Aichi, Japan) [36], and the serum levels RF and CRP that were measured with latex particle-enhanced and simple nephelometric methods (SRL, Tokyo, Japan), respectively. Positivity for anti-CCP IgG and RF was defined as measurements more than 4.5 U/mL and 15 IU/mL, respectively.

### Evaluation of serum CarP and NETs levels

To evaluate the serum CarP levels, the quantity of carbamylated adduct in the serum samples was determined by a sandwich ELISA with a commercially available kit (Cell Biolabs Laboratories, INC. San Diego, CA) [37] according to the manufacturer’s instructions. For the evaluation of serum NETs levels, NETs-associated MPO-DNA complexes were quantified by an ELISA as described previously [38]. The brief protocol of detection for MPO-DNA complexes was as follows: 96-well microtiter plates were coated with 5 μg/ml of mouse anti-human MPO antibody (Bio-Rad Laboratories, Hercules, CA) overnight at 4°C, blocked with 1% bovine serum albumin for 30 min at room temperature (RT), washed three times, incubated with serum samples (1:10 dilution) for 90 min at RT, washed three times, incubated with horseradish peroxidase-labeled anti-human DNA monoclonal antibody (Roche Diagnostics, Mannheim, Germany) for 90 min at RT, and underwent color development with 2,2'-Azino-bis (3-ethylbenzothiazoline-6-sulfonic acid) diammonium salt substrate for 20 min at RT, at which point the reaction was stopped. The optical absorbance was measured with a microplate reader (EMax Plus, Molecular Devices, LLC. Sunnyvale, CA) at 450 nm for CarP or at 405 nm for MPO-DNA complexes. Serum levels of CarP and NETs were expressed as ng/mL and ELISA units (EU), respectively.

### Statistical analyses

The sample size calculation was performed using the SPSS software program (SamplePower version 3.0, IBM, Chicago, IL) based on the serum citrulline levels shown in a previous study [27]. The results revealed that having more than 20 patients in each group would exceed a statistical power of 0.8 with an alpha level of 5%. After evaluating the normality of distribution by Kolmogorov-Smirnov test, the differences in quantitative data among the groups were assessed by the Kruskal-Wallis test, followed by Scheffe’s test. Spearman’s rank correlation coefficient was used to determine the relationship between the serum levels of CarP and NETs and RA activity or periodontitis severity (mean probing depth and mean clinical attachment level) and among the serum levels of CarP, NETs, and anti-CCP IgG. Furthermore, the associations between the serum levels of CarP and NETs and periodontitis severity (healthy and mild periodontitis coded as 0, and moderate and severe periodontitis coded as 1) were assessed by multiple logistic regression analyses using a statistical software program (SPSS statistics version 21, IBM Japan, Tokyo, Japan). The possible covariates considered for the multiple logistic regression analyses were age, gender (male and female coded as 0 and 1), smoking status (never-smoker, former-smoker, and current-smoker coded as 0, 1, and 2), and the odds ratio (OR) and 95% confidence interval (95% CI) were calculated. The differences in quantitative data between the baseline and reassessment were assessed by Mann Whitney *U*-test. Statistical significance was considered to exist at 5% (P < 0.05).

## Results

### Comparison among the groups

**Fig 2:**
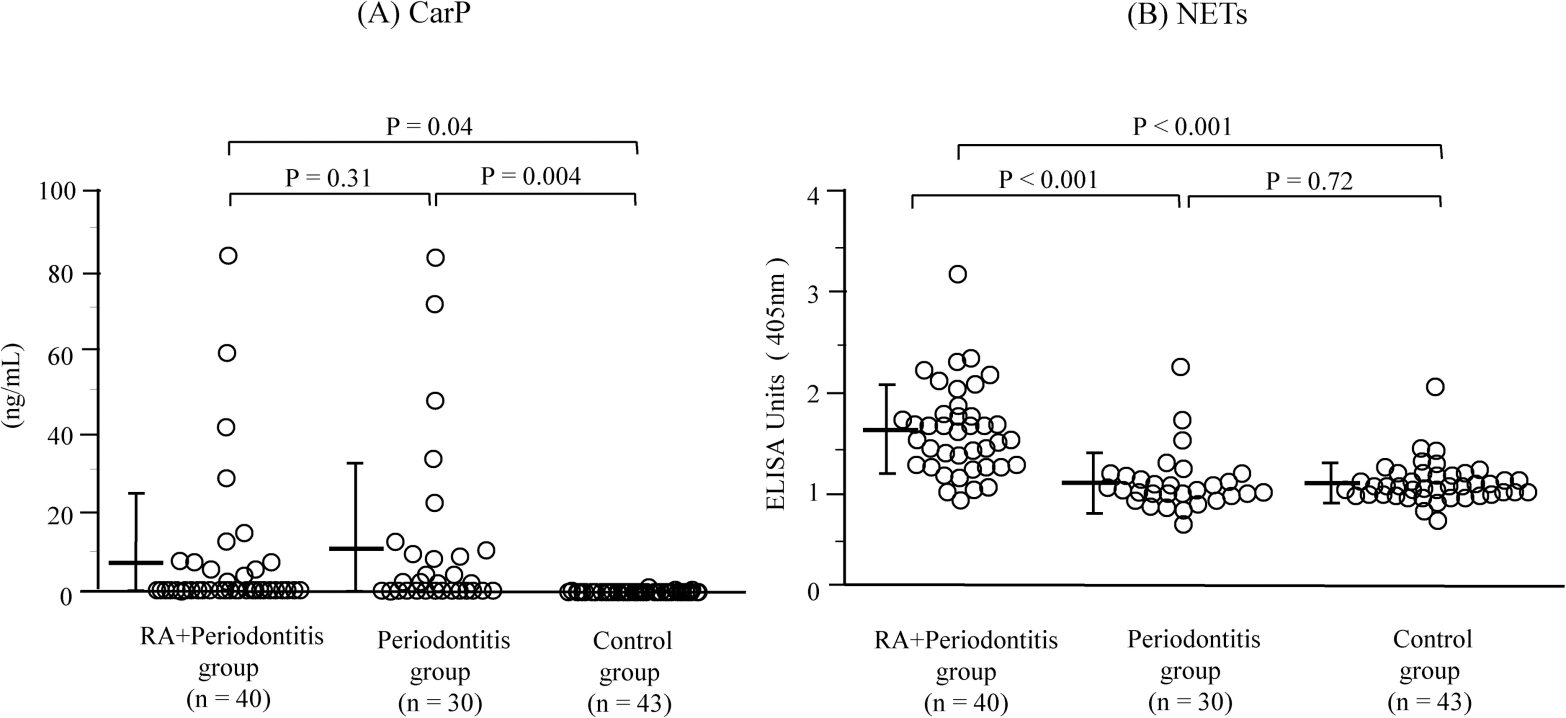
**Serum levels of (A) CarP and (B) NETs in patients with RA and periodontitis, in patients with periodontitis, and in the controls.** Horizontal bars indicate the mean and SD.

**Fig 3:**
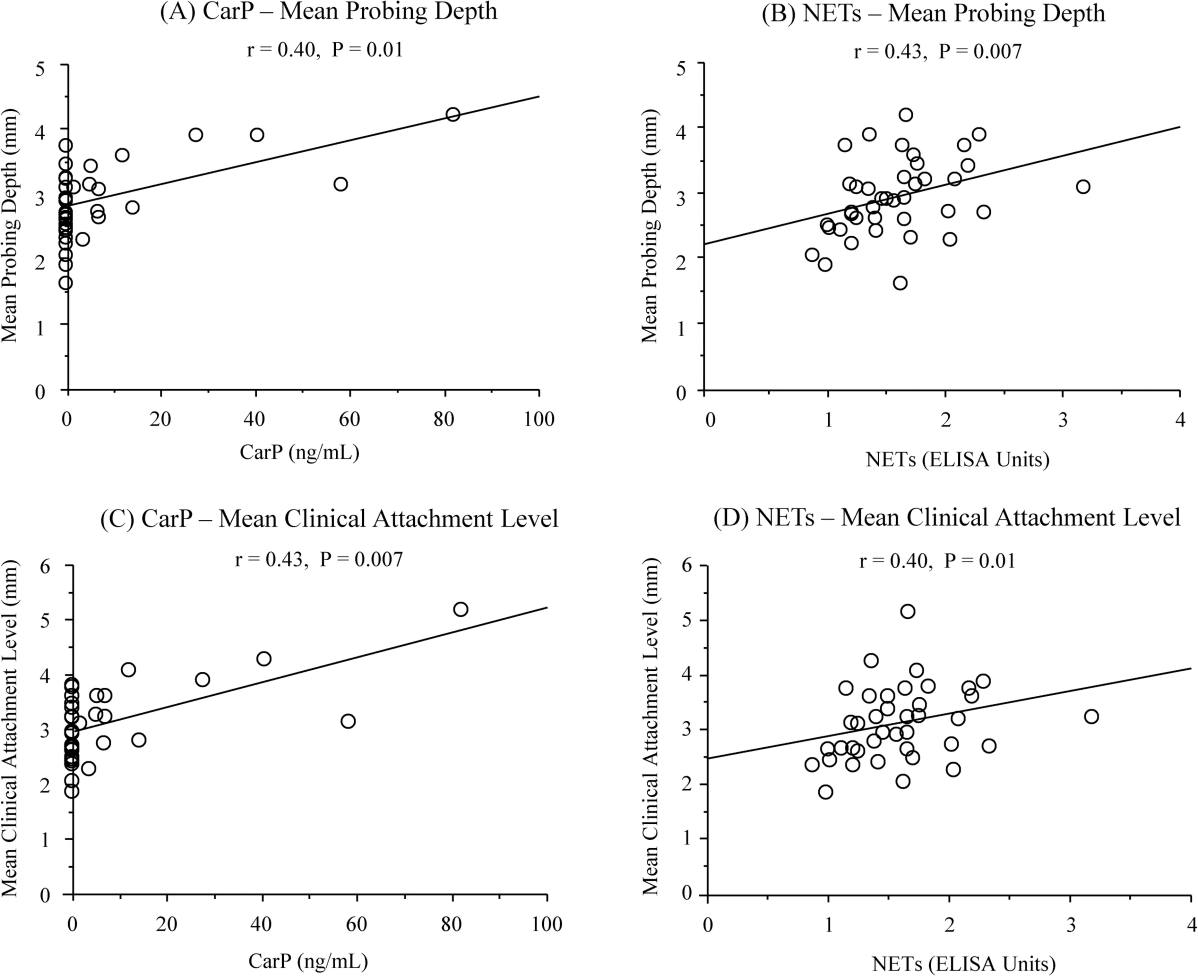
**Relationship between serum CarP and NETs levels and mean values of probing depth and clinical attachment level in 40 patients with RA and periodontitis (A to D)**.

**Fig 4:**
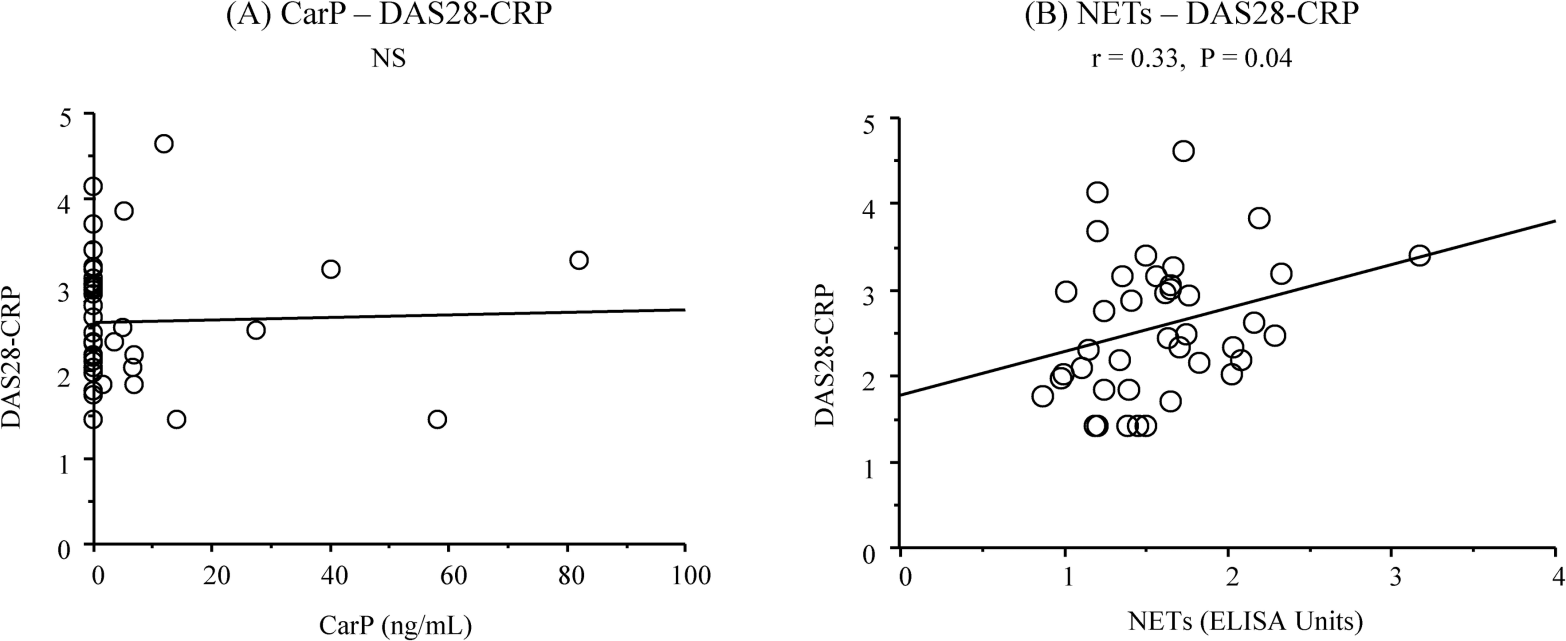
**Relationship between serum CarP and NETs levels and DAS28-CRP in 40 patients with RA and periodontitis (A and B).** NS: no significant association.

**Fig 5:**
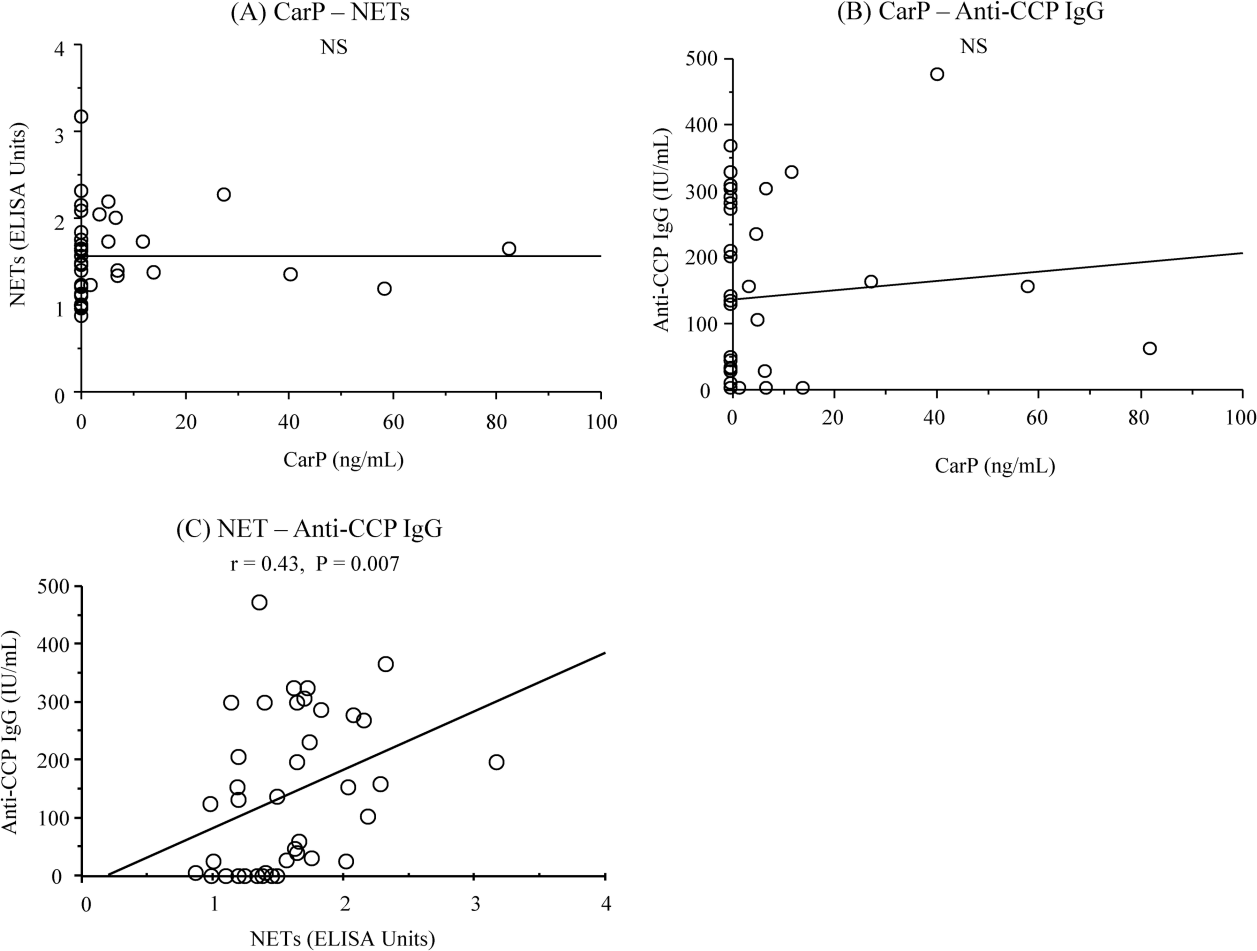
**Relationship among serum levels of CarP, NETs, and anti-CCP IgG in 40 patients with RA and periodontitis (A to C).** NS: no significant association.

The characteristics of the study population are presented in Table 1. No significant differences were observed among the groups in any demographic parameter values (P > 0.05) (Table 1). When compared to the control group, the RA+periodontitis group displayed significantly fewer teeth (P = 0.01) (Table 1), and both the RA+periodontitis and periodontitis groups showed significantly higher values of plaque control record, bleeding on probing, probing depth, and clinical attachment level as well as higher percentage of patients with moderate and severe periodontitis (P < 0.001 for all parameters) (Table 1). The RA+periodontitis group received medication with corticosteroids, non-steroidal anti-inflammatory drugs (NSAIDs), and conventional synthetic disease-modifying antirheumatic drugs (csDMARDs) but not with any biological DMARDs (bDMARDs), including inhibitors of tumor necrosis factor, interleukin-6 receptor, and the co-stimulatory signal for T cell activation (Table 1). Univariate analyses revealed significantly higher serum CarP levels in the RA+periodontitis and periodontitis groups than in the control group (RA+periodontitis and periodontitis versus control: [mean ± standard deviation] 6.9 ± 17.2 ng/mL and 10.4 ± 20.8 ng/mL versus 0.1 ± 0.2 ng/mL, P = 0.04 and P = 0.004) (Fig 2A). The frequencies of individuals who had CarP in the serum samples were 32.5% in the RA+periodontitis group, 53.3% in the periodontitis group, and 7.0% in the control group. The serum NETs levels were significantly increased in the RA+periodontitis group compared to the periodontitis and control groups (RA+periodontitis versus periodontitis and control: 1.6 ± 0.5 EU versus 1.1 ± 0.3 EU and 1.0 ± 0.2 EU, P < 0.001 for both comparisons) (Fig 2B). The serum levels of both CarP and NETs were significantly correlated positively with the mean values of probing depth (P = 0.01 and P = 0.007, respectively) and those of the clinical attachment level (P = 0.007 and P = 0.001, respectively) in the 40 patients with RA and periodontitis (Fig 3A to 3D). Multiple logistic regression analyses also revealed that the serum levels of CarP and NETs were significantly associated with moderate to severe periodontitis (CarP: P = 0.03, OR = 2.05, 95% CI = 1.09 to 3.85; NETs: P = 0.001, OR = 14.49, 95% CI = 2.99 to 71.43) (Table 2). However, a significantly positive correlation was observed between the serum NETs levels and DAS28-CRP (P = 0.04) but not between the serum CarP levels and DAS28-CRP (P > 0.05) (Fig 4A and 4B). Furthermore, the serum CarP levels were not associated with the serum levels of NETs and anti-CCP IgG (P > 0.05) (Fig 5A and 5B), while the serum NETs levels were significantly correlated positively with the serum anti-CCP IgG levels (P = 0.007) (Fig 5C).

**Table 1:** Characteristics of the study population.

Parameter	RA+Periodontitis group (n = 40)	Periodontitis group (n = 30)	Control group (n = 43)	P-value
Demographic				
Age, mean (SD) years	59.9 (13.3)	55.5 (10.8)	57.6 (9.5)	0.15
Female, no. (SD) %	34 (85.0)	25 (83.3)	37 (86.0)	0.95
Smoker of current/former/never, %	0/35/65	0/30/70	0/16/84	0.14
Periodontal				
Teeth present, no. (SD)	24.9 (2.6)*	25.4 (2.8)	26.5 (2.2)	0.01
Plaque control record, mean (SD) %	51.7 (21.5)*	57.7 (16.5)*	17.3 (17.6)	< 0.001
Bleeding on probing, mean (SD) %	15.8 (11.5)*	18.1 (15.1)*	3.6 (6.3)	< 0.001
Probing depth, mean (SD) mm	2.9 (0.6)*	2.9 (0.5)*	2.2 (0.4)	< 0.001
Probing depth ≥ 4 mm, mean (SD) %	22.6 (21.2)*	23.1 (9.4)*	2.6 (0.3)	< 0.001
Clinical attachment level, mean (SD) mm	3.1 (0.7)*	3.2 (0.6)*	2.3 (0.4)	< 0.001
Clinical attachment level ≥ 4 mm, mean (SD) %	26.1 (23.2)*	28.6 (9.4)*	2.2 (0.4)	< 0.001
Periodontitis of no/mild/moderate/severe ^a^, %	0/20/33/47*	0/0/47/53*	100/0/0/0	< 0.001
Rheumatologic				
Disease duration, mean (SD) months	12.0 (11.3)	N/A	N/A	
DAS28-CRP, mean (SD)	2.6 (0.8)	N/A	N/A	
Remission/low/moderate/high activity (%)	40/18/37/5	N/A	N/A	
Tender joint count, mean (SD)	1.1 (1.7)	N/A	N/A	
Swollen joint count, mean (SD)	1.6 (2.3)	N/A	N/A	
VAS, mean (SD) mm	27.5 (26.5)	N/A	N/A	
Corticosteroid use, no. (%)	21 (52.5)	N/A	N/A	
csDMARD use, no. (%)	28 (70.0)	N/A	N/A	
bDMARD use, no. (%)	0 (0.0)	N/A	N/A	
NSAID use, no (%)	7 (17.5)	N/A	N/A	
Anti-CCP IgG level, mean (SD) U/mL	139.7 (133.9)	N/A	N/A	
Anti-CCP IgG positive, no. (%)	31 (77.5)	N/A	N/A	
RF level, mean (SD) IU/mL	70.6 (85.3)	N/A	N/A	
RF positive, no. (%)	25 (62.5)	N/A	N/A	
CRP level, mean (SD) mg/dL	0.4 (0.6)	N/A	N/A	

RA: rheumatoid arthritis; SD: standard deviation; N/A: not applicable; DAS28-CRP: disease activity score including 28 joints using C-reactive protein; VAS: visual analog scale; csDMARD: conventional synthetic disease-modifying antirheumatic drug; bDMARD: biological disease-modifying antirheumatic drug; NSAID: non-steroidal anti-inflammatory drug; CCP: cyclic citrullinated peptide; IgG: immunoglobulin G; RF: rheumatoid factor; EU: ELISA unit.

^a^ Periodontitis case was defined according to the criteria of the Centers for Disease Control and Prevention /American Academy of Periodontology [30].

* The bold values are significantly different from those in the control group (P < 0.05).

**Table 2:** Significance of association between the baseline characteristics and periodontitis severity in the study population (n = 113).

Parameter	OR (95% CI)	P-value
Age	1.02 (0.98 to 1.07)	0.32
Women	1.17 (0.24 to 5.71)	0.85
Former smoker	2.91 (0.75 to 11.24)	0.12
Serum CarP level	2.05 (1.09 to 3.85)	0.03*
Serum NETs level	14.49 (2.99 to 71.43)	0.001*

CarP: carbamylated protein; NETs: neutrophil extracellular traps.

* Significantly associated with moderate and severe periodontitis (P < 0.05) (bold value).

### Comparison before and after periodontal treatment

**Fig 6:**
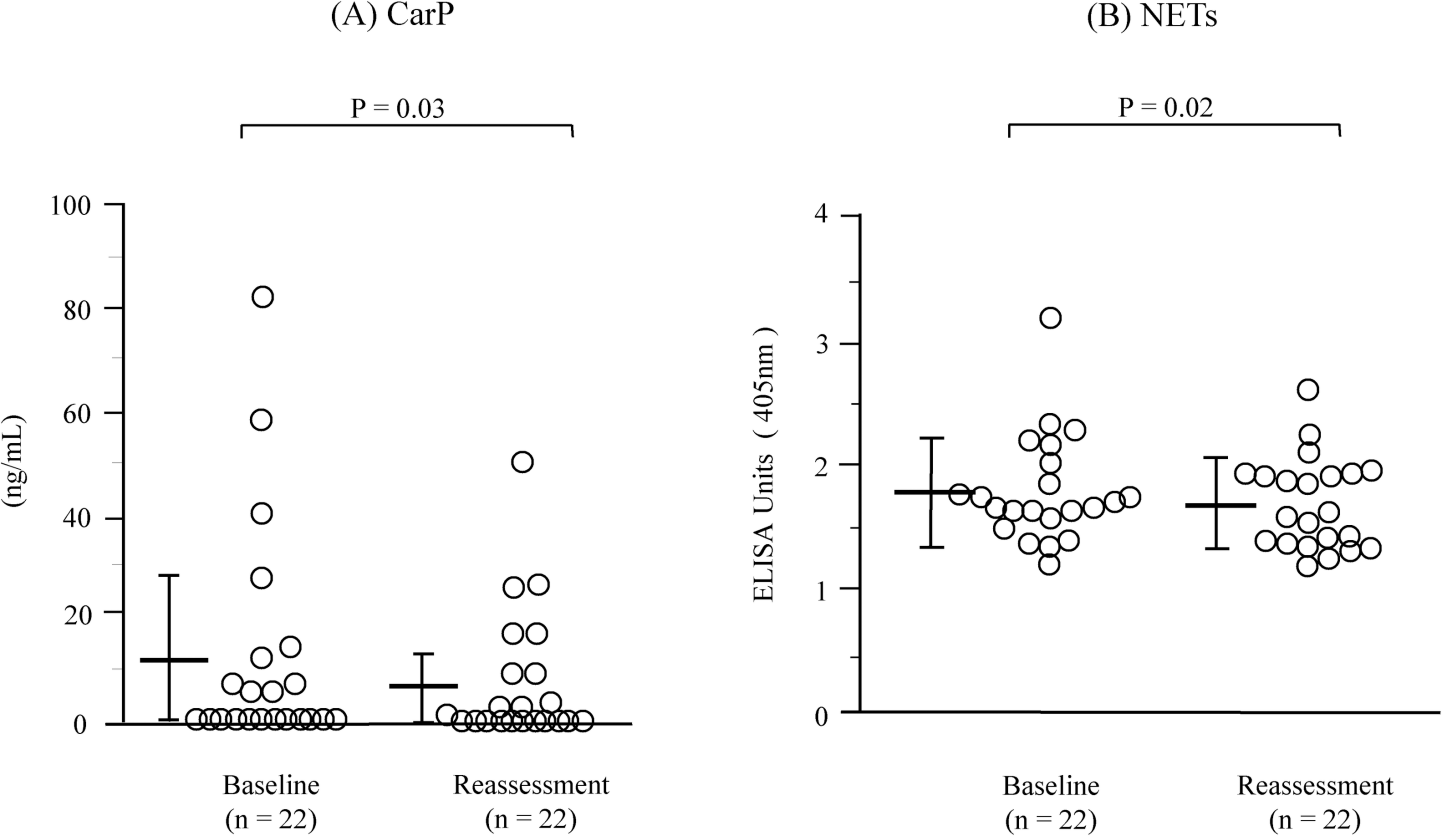
**Comparison of serum levels of (A) CarP and (B) NETs between the baseline and reassessment in patients with RA and periodontitis who received periodontal treatment.** Horizontal bars indicate the mean and SD.

In the 22 patients in the RA+periodontitis group who had received periodontal treatment, the distribution and dose of RA medication with corticosteroids, NSAIDs, or csDMARDs remained unchanged between the baseline and reassessment. Periodontal treatment significantly decreased the values of periodontal parameters, including the plaque control record, bleeding on probing, probing depth, clinical attachment level, and percentage of patients with severe periodontitis (P < 0.001 for all parameters), as well as the DAS28-CRP and percentage of patients with moderate RA activities (P = 0.02 and P = 0.04) (Table 3). In addition, the treatment significantly reduced the serum levels of CarP and NETs (baseline versus reassessment: 11.7 ± 21.8 ng/mL versus 7.1 ± 12.9 ng/mL, P = 0.03 for CarP; 1.8 ± 0.4 EU versus 1.7 ± 0.4 EU, P = 0.02 for NETs) (Fig 6A and 6B).

**Table 3:** Comparison of periodontal and rheumatologic parameters between the baseline and reassessment in patients with RA and periodontitis who received periodontal treatment.

Parameter	Baseline (n = 22)	Reassessment (n = 22)	P-value
Demographic			
Age, mean (SD) years	60.3 (14.4)	60.3 (14.4)	ND
Female, no. (SD) %	18 (81.8)	18 (81.8)	ND
Smoker of current/former/never, %	0/41/59	0/41/59	ND
Periodontal			
Teeth present, no. (SD)	24.7 (2.7)	24.7 (2.7)	ND
Plaque control record, mean (SD) %	58.6 (21.9)	25.5 (17.4)*	< 0.001
Bleeding on probing, mean (SD) %	15.7 (12.5)	5.9 (5.6)*	< 0.001
Probing depth, mean (SD) mm	3.2 (0.6)	2.8 (0.7)*	< 0.001
Probing depth ≥ 4 mm, mean (SD) %	31.2 (22.9)	14.6 (21.3)*	< 0.001
Clinical attachment level, mean (SD) mm	3.3 (0.7)	2.9 (0.8)*	< 0.001
Clinical attachment level ≥ 4 mm, mean (SD) %	36.6 (24.6)	19.2 (22.6)*	< 0.001
Periodontitis of no/mild/moderate/severe ^a^, %	0/4/41/55	4/32/50/14*	< 0.001
Rheumatologic			
Disease duration, mean (SD) years	11.8 (11.7)	11.8 (11.7)	ND
DAS28-CRP, mean (SD)	2.7 (0.8)	2.3 (0.7)*	0.02
Remission/low/moderate/high activity (%)	27/23/45/5	50/23/23/4*	0.04
Tender joint count, mean (SD)	1.3 (1.9)	1.2 (1.9)	0.85
Swollen joint count, mean (SD)	1.8 (2.6)	1.2 (1.4)	0.30
VAS, mean (SD) mm	31.0 (25.8)	30.3 (22.7)	0.81
Corticosteroid use, no. (%)	13 (59.1)	13 (59.1)	ND
csDMARD use, no. (%)	15 (68.2)	15 (68.2)	ND
bDMARD use, no. (%)	0 (0.0)	0 (0.0)	ND
NSAID use, no (%)	4 (18.2)	4 (18.2)	ND
Anti-CCP IgG level, mean (SD) U/mL	163.7 (139.7)	164.2 (125.5)	0.14
Anti-CCP IgG positive, no. (%)	20 (90.9)	20 (90.9)	ND
RF level, mean (SD) IU/mL	65.5 (77.4)	68.2 (87.0)	0.76
RF positive, no. (%)	15 (68.2)	15 (68.2)	ND
CRP level, mean (SD) mg/dL	0.4 (0.7)	0.4 (0.7)	0.96

RA: rheumatoid arthritis; SD: standard deviation; ND: not determined; DAS28-CRP: disease activity score including 28 joints using C-reactive protein; VAS: visual analog scale; csDMARD: conventional synthetic disease-modifying antirheumatic drug; bDMARD: biological disease-modifying antirheumatic drug; NSAID: non-steroidal anti-inflammatory drug; CCP: cyclic citrullinated peptide; IgG: immunoglobulin G; RF: rheumatoid factor; EU: ELISA unit.

^a^ Periodontitis case was defined according to the criteria of the Centers for Disease Control and Prevention /American Academy of Periodontology [30].

* The bold values are significantly different from the baseline values (P < 0.05).

## Discussion

The present pilot case-control study aimed to test the hypothesis that protein carbamylation and NETs formation in patients with RA may also be shared by periodontitis. The results of univariate analyses showed that the patients with RA and periodontitis exhibited significantly higher serum levels of CarP and NETs than the age-, gender-, smoking status-matched controls. These results suggest that the circulating levels of CarP and NETs were associated not only with RA but also periodontitis, which is supported by the findings described in other reports [39, 40]. In addition, the data indicated that the serum levels of CarP and NETs were significantly correlated positively with the mean values of the probing depth and clinical attachment level in patients with RA and periodontitis. Furthermore, the results of the multiple regression analysis demonstrated that the serum levels of CarP and NETs were significantly associated with moderate to severe periodontitis. These findings suggest a positive association between the circulating levels of CarP and NETs and periodontitis severity. The elevation of circulating levels of CarP and NETs may reflect the increased concentration of MPO in the sera, salivary, gingival crevicular fluid, and periodontal tissue of in patients with periodontitis [39, 40]. To our knowledge, this is the first study to show that the circulating levels of CarP and NETs constitute a shared risk factor for RA and periodontitis, which may provide new insights into the underlying shared pathogenic mechanisms of RA and periodontitis.

Another objective of the present study was to evaluate the effect of periodontal treatment on the serum levels of CarP and NETs in patients with RA and periodontitis. The present results showed that periodontal treatment significantly decreased serum levels of CarP and NETs. The decrease in serum NETs levels is consistent with the results of another study that examined the changes in the NETs production derived from blood neutrophils before and after periodontal therapy [25]. These observations may reflect reduced levels of priming of circulating neutrophils after successful periodontal treatment. Likewise, the post-treatment decrease in the serum CarP levels might be due in part to the reduced release of MPO from neutrophils, as protein carbamylation is catalyzed by MPO [11]. The data also showed that the levels of periodontal tissue destruction, as determined by the probing depth and clinical attachment level, in patients with RA were significantly reduced by periodontal treatment. These observations support the possibility that circulating levels of CarP and NETs can constitute a novel biomarker associated with periodontitis severity in patients with RA and periodontitis. Furthermore, the results showed that periodontal treatment significantly decreased RA activity, which is consistent with the findings of our previous and another study [8, 27] which suggested the existence of an interrelationship between RA and periodontitis.

Interestingly, the results of the current study also indicated that the patients with only periodontitis had significantly higher circulating levels of CarP than the controls, which is supported by the findings of another study that showed the presence of CarP in inflamed periodontal tissue [17]. However, the serum NETs levels were comparable between the patients with periodontitis and controls in the present study. These are consistent with the results of another study [25], which suggests the possibility of a recently reported self-protective mechanism within glutathione-deficient periodontitis neutrophils [41].

Another interesting note is that the serum NETs levels were significantly correlated with the RA activity in patients with RA and periodontitis. These findings are supported by the observations of another study that documented a potential association between NETs-derived products, including MPO and RA activity [42]. However, the results failed to show an association between the serum CarP levels and RA activity. The additional data indicated a significantly positive correlation between the serum levels of NETs and anti-CCP IgG in patients with RA and periodontitis. These results suggest that NETs may be a source of citrullinated proteins, and that elevated levels of circulating NETs may play a role in the progression of RA through protein citrullination. However, the data showed that the serum CarP levels were not associated with the serum levels of NETs and anti-CCP IgG. These observations suggest that NETs may not be a source of carbamylated proteins, which may be explained in part by the fact that many patients with RA and periodontitis exhibited no serum CarP.

The present study has some methodological limitations. First, the sample size was relatively small due to the strict eligibility criteria, resulting in this being only a pilot case-control study. Second, RA and periodontitis are complex diseases with multiple causal factors [1, 3, 5]. However, controlling for the confounding factors was restricted in the statistical analysis, as information on the risk factors was not fully available. Third, case-control studies are associated with some selection bias. However, data on the mean age and the percentage of females among patients with RA in the present study were similar to those of other large-scale race-matched cohort studies [35, 43], suggesting that the selection bias was minimal, although its possibility cannot be excluded. Fourth, we were unable to evaluate anti-CarP antibody and MPO due to the very limited amount of serum sample available. However, these evaluations will be necessary to validate whether or not RA and periodontitis have a specific interrelationship through protein carbamylation and NETs formation. Finally, we were unable to recruit any patients with RA who did not have periodontitis, possibly due to their extremely low numbers [8, 44]. However, it would be preferable to assess the shared risk factor for the two diseases in a study population including individuals with both diseases, individuals with either disease, and controls. As such, further studies in a large-scale cohort are required to confirm and extend the current observations.

In conclusion, the results of the present pilot case-control study showed for the first time that the circulating levels of CarP and NETs are associated with periodontitis severity and influenced by periodontal treatment in patients with RA. These data may provide new insights into the underlying shared pathogenic mechanisms of RA and periodontitis. Further replication studies in a large-scale cohort are needed to confirm the current observations.

## Supporting information

S1 Table STROBE Checklist for the Case-Control study. (DOCX)
