# Supplementary material for: Circulating levels of carbamylated protein and neutrophil extracellular traps are associated with periodontitis severity in patients with rheumatoid arthritis: A pilot case-control study
Source: PLoS One. 2018 Feb 2;13(2):e0192365. doi: 10.1371/journal.pone.0192365 (PMC5796721; doi:10.1371/journal.pone.0192365)
Supplement: S1 Table — (DOCX) [file pone.0192365.s001.docx]

Dear the PLOS ONE editorial staff,

Thank you for your e-mail information (dated on Jan 25, 2018) on required modifications for our manuscript (PONE-D-17-43688R1).

In response to 4 points journal requirements, I have now completed the confirmation and correction, which has now been listed below.

1. Please ensure that the author list and affiliations are correct on the title page of your manuscript, and that your author contributions, competing interests, and financial disclosure are correct as listed below.

Answer: I have now found some errors in the contributor roles listed in your e-mail, and have informed you of the corrected changes (red characters) as listed below, to match with the Author Contributions in the revised manuscript. In addition, I have now confirmed that the author list and affiliations on the title page, the competing interests, and financial disclosure (funding information) are correct.

Tetsuo Kobayashi

Conceptualization

Data curation

Formal analysis

Funding acquisition

Investigation

Methodology

Project administration

Resources

Software

Writing – original draft

Chihiro Kaneko

Data curation →　please delete

Formal analysis

Investigation

Methodology →　please delete

Writing – original draft

Satoshi Ito            please add “Resources”

Data curation →　please delete

Formal analysis →　please delete

Investigation →　please delete

Methodology

Writing – original draft

Noriko Sugita      please add “Resources”

Data curation

Methodology →　please delete

Akira Murasawa  please add “Resources”

Data curation →　please delete

Methodology →　please delete

Kiyoshi Nakazono  please add “Resources”

Data curation →　please delete

Methodology →　please delete

Hiromasa Yoshie

Supervision

1. Please confirm that all information in your Funding Information is also present in your Financial Disclosure.

Answer: I have now confirmed that all of these are correct.

1. Tables 1 and 3 contain hard returns to align text, which PLOS does not allow. Within tables, there can be no picture elements, text boxes, tabs, spaces used to create line breaks, returns (i.e. clicking "Enter" or "Return" at the end of a line) to align text within a cell, or vertical text. You may use returns to format a list within a cell, however, you may not use hard or soft returns to achieve alignment across multiple cells in a row, as this can cause the planned alignment to be thrown off during typesetting. Please reformat your table(s) according to our requirements.

Answer: I have now conducted the reformation of Tables 1 and 3, according to your request.

1. Thank you for including your figure captions and tables in read-through order within your manuscript text. Could you please create line breaks/spaces (press the "enter" key once or twice) between your figure captions and your table footnotes and the manuscript text so that our typesetter can correctly see which text should be your figure caption and which text should be your main text?

Answer: I have now created line breaks/spaces, according to your request.

Please let me know immediately if you have any troubles with this matter.

Thank you for your kind consideration,

With kind regards,

Tetsuo Kobayashi

Niigata University
